# Dynamic needle tip positioning in short-axis versus long-axis ultrasound-guided arterial cannulation: a randomised controlled trial

**DOI:** 10.1186/s12871-025-03413-3

**Published:** 2025-11-14

**Authors:** Maram I. Elmazny, Sondos Ahmed, Sarah Afifi

**Affiliations:** 1 Anesthesiology, Intensive Care and Pain Medicine Department, Faculty of Medicine, Tanta University, Tanta, Egypt; 2 Anesthesiology, Intensive Care and Pain Department, Faculty of Medicine, Ain Shams University, Cairo, Egypt; 3 Lecturer of Anesthesiology, Intensive Care and Pain Medicine Department, Faculty of Medicine, Tanta University, Tanta, 31511 Egypt

**Keywords:** Arterial cannulation, Dynamic needle tip positioning, Short-Axis, Long-Axis, Ultrasound, Radial artery

## Abstract

**Background:**

Advancements in ultrasound-guided arterial cannulation (USG-AC) are ongoing, with evidence supporting the clinical efficacy of both short-axis (SA) and long-axis (LA) approaches. The objective was to ascertain the efficacy of dynamic needle tip positioning (DNTP) modification in SA versus LA approaches for USG-AC.

**Methods:**

In this prospective randomised controlled trial, 164 individuals were scheduled for elective surgery requiring AC. Randomisation allocated cases to DNTP, which were received as either SA or LA. The procedure involved precise patient positioning, sterile technique, and USG.

**Results:**

SA group demonstrated significantly shorter US location times (6.16 ± 1.99 vs. 13.65 ± 3.71 s, *p* < 0.001) and cannulation times (10.41 ± 3.25 vs. 29.41 ± 5.27 s, *p* < 0.001) compared to LA group. First-pass success rates were higher in the SA group (92.68% vs. 80.49%, *p* = 0.022). Complication rates were comparable between groups, with no thrombosis or nerve injury cases. Operator satisfaction was notably greater in the SA group (*p* = 0.007) than in the LA group.

**Conclusions:**

With DNTP modification, the SA approach demonstrates superior efficacy and success rates than the LA approach for USG-AC while maintaining comparable safety profiles and higher operator satisfaction.

**Trial registration:**

registering on ClinicalTrials.gov (ID: NCT06422195) URL: https://clinicaltrials.gov/study/NCT06422195?cond=NCT06422195&rank=1 (Date: 2024-05-20).

## Background

In critical care units, emergency departments, and surgical suites, arterial cannulation (AC) is principally employed when a necessity for uninterrupted blood pressure surveillance and regular assessment of blood gases [1, 2]. This procedure has become increasingly vital, particularly in high-risk patients and cases involving anticipated major fluid shifts [3]. Due to its superficial location, collateral circulation through the ulnar artery, and a minimal incidence of adverse events, AC protocols frequently prioritise the radial artery (RA) as the primary insertion site [4].

The incidence of frequently encountered complications associated with AC, including thrombus formation, hematoma development, localised oedema, and vasospasm, tends to rise with repeated failed cannulation attempts; these repeated attempts also heighten the likelihood of arterial spasms and patient distress, thus motivating the creation of refined techniques designed to improve procedural efficacy [5, 6].

The integration of ultrasound (US) guidance has significantly advanced AC procedures, particularly by enhancing first-attempt success rates in RA cannulation with real-time US guidance [7]. Two primary US-guided approaches have been established in clinical practice: the short-axis (SA) out-of-plane and the long-axis (LA) in-plane techniques [8–10].

A clear visual representation of significant anatomical elements, encompassing vascular structural variations, is rendered possible through the SA approach, nerve pathways, and neighbouring veins throughout the cannulation process. In contrast, utilising the LA approach offers an enhanced view of the needle’s path as it nears and penetrates the arterial lumen, which may increase the likelihood of successful catheter placement [11, 12].

Dynamic needle tip positioning (DNTP), a significant innovation in US-guided cannulation, conceptually integrates the benefits of both conventional methods, suggesting an improvement in the likelihood of successful cannulation on the initial attempt [13]. This adapted SA method utilises a stepwise needle progression coupled with a simultaneous proximal movement of the US transducer, and the needle is inserted incrementally until its distal end is no longer discernible via the US [14].

While both methods demonstrate clinical efficacy, continued research into optimal training strategies and implementation protocols remains essential for advancing practice standards in critical care settings [15]. Clinical experience with DNTP modification for US-guided AC is limited, with few studies employing this technique.

We hypothesise that DNTP modification in the SA approach provides a faster USG-AC than the LA approach. This study aims to compare the efficacy of DNTP in SA and LA approaches for USG-AC.

## Methods

A randomised, prospective clinical trial conducted at Tanta University Hospitals (May-December 2024) included 164 patients (age 18–70, ASA II-IV) undergoing elective surgery requiring invasive arterial pressure monitoring.

The research was carried out after receiving approval from the Ethics Committee (approval code: 36197/12/22), this study was done in accordance with declaration of Helisinki (2013) and registering on ClinicalTrials.gov (ID: NCT06422195) URL: https://clinicaltrials.gov/study/NCT06422195?cond=NCT06422195&rank=1 (Date: 2024-05-20), and all participants gave informed consent.

Exclusion criteria were applied based on the following criteria: emergent circumstances, hemodynamic instability, evidence of cellulitis or infection at the proposed cannulation site, a positive result on the modified Allen test, diagnosis of Raynaud disease or peripheral vascular disease, more than one RA procedure within the preceding 30 days, scheduled surgery at the intended cannulation site, or refusal to participate.

### Randomisation and blindness

Equal randomisation (1:1), utilising (https://www.randomizer.org/) and sealed opaque envelopes, allocated participants (*n* = 164) into two groups (*n* = 82 per group) to receive DNTP (SA or LA); blinding of participants and outcome assessors were implemented. Different anesthesiologists performed procedures for each technique. Both operators were experienced (> 50 supervised procedures each).

A comprehensive preoperative evaluation was conducted to ensure patient readiness for the procedure. This included documenting the patient’s medical history, performing a detailed clinical examination, and carrying out standard laboratory investigations. All patients received thorough counselling about the procedure, addressing potential risks and expected outcomes.

Standard monitoring protocols were implemented to ensure patient safety upon arrival in the operating theatre. These included the attachment of an electrocardiogram, the establishment of non-invasive blood pressure monitoring, and the connection of pulse oximetry. An 18-gauge intravenous cannula was inserted, then midazolam (0.05 mg/kg) was administered to each patient.

The RA cannulation procedure required precise anatomical positioning. Mild wrist extension was achieved using a cylindrical towel roll, and the arm was positioned with a slight degree of abduction. The extremity was then secured to an armboard using medical tape. To ensure an aseptic environment, sterile procedures were implemented throughout the intervention, incorporating drapes, sterile gloves, and US probe covers. The Philips eL18-4 PureWave Linear Array Transducer was utilised, operating within a frequency range of 2 to 22 MHz, with an optimised range of 4 to 18 MHz.

### DNTP-SA

An experienced anesthesiologist, uninvolved in further aspects of the study, performed the procedure using a SA approach. To achieve a transverse view, the perpendicular positioning of the US transducer relative to the RA enabled the visualisation of the vessel as a circular, anechoic structure on the display. Simultaneous proximal movement of the US probe accompanied progressive needle advancement; the cannula was then advanced into the arterial lumen after the US screen no longer displayed the needle tip.

### DNTP-LA

Another anesthesiologist, uninvolved in subsequent study assessments, performed the procedure using the LA approach. The US probe was positioned parallel to the RA, allowing visualisation of the vessel as a tubular anechoic structure. Continuous USG was employed to achieve direct cannulation.

Procedural timing began with the initiation of US scanning and ended upon detecting an arterial waveform on the monitoring device; achieving an arterial waveform after a solitary skin puncture was considered a first-pass success. If RA cannulation remained unsuccessful after five minutes, the procedure was concluded, and alternative methods were implemented to maintain patient well-being.

The modified DNTP-SA technique involves perpendicular probe placement to the artery and synchronised 1–2 mm probe (rather than 3–5 mm) and needle advancements for continuous needle-tip visualisation, reducing complications (reduce arterial wall indentation and minimise posterior wall penetration). In DNTP-LA, the probe aligns parallel to the artery, with in-plane needle guidance and frequent alignment checks, adapting DNTP principles for improved accuracy and safety.

The study documented various procedural metrics, encompassing anatomical dimensions like the anteroposterior diameter of the RA and the distance from the skin surface to the anterior arterial wall; temporal data, such as the duration of US localisation and the time taken for successful cannulation in seconds; and technical aspects, including the rate of first-pass success and the total number of skin punctures.

Hemodynamic data, including systolic, diastolic, and heart rate, were documented before and after the procedure.

Complications were assessed for oedema, hematoma formation, vasospasm, ischemic events, thrombosis, and nerve injury.

The study assessed operator satisfaction utilising a five-point Likert scale [16], where 1 indicated “Very Unsatisfied,” 2 corresponded to “Unsatisfied,” 3 represented “Neutral,” 4 denoted “Satisfied,” and 5 signified “Very Satisfied.

The primary outcome is the time to achieve successful cannulation (defined as the duration from initial skin puncture to confirmation of arterial waveform on the monitor). Secondary outcomes include US location time, first-pass success rate, number of punctures required, the frequency of complications, and the level of satisfaction reported by the operators.

### Sample size calculation

A sample of 82 subjects for each group was determined using G*Power 3.1.9.2, with considerations for dropout, based on an effect size of 0.6, 95% confidence, and 95% power, which were derived from initial pilot data (*n* = 10) that showed a mean cannulation time disparity of 3 s between the SA (14.4 ± 1.5s) and LA (17.4 ± 6.98s) cohorts.

### Statistical analysis

Utilising SPSS version 27 (IBM^©^, Armonk, NY, USA), data normality was evaluated with Shapiro-Wilk tests and histograms, parametric data (mean ± SD) were compared via unpaired t-tests, and categorical variables (frequencies/percentages) were examined with Chi-square or Fisher’s exact tests, with significance two-tailed defined as *p* < 0.05.

## Results

**Fig. 1:**
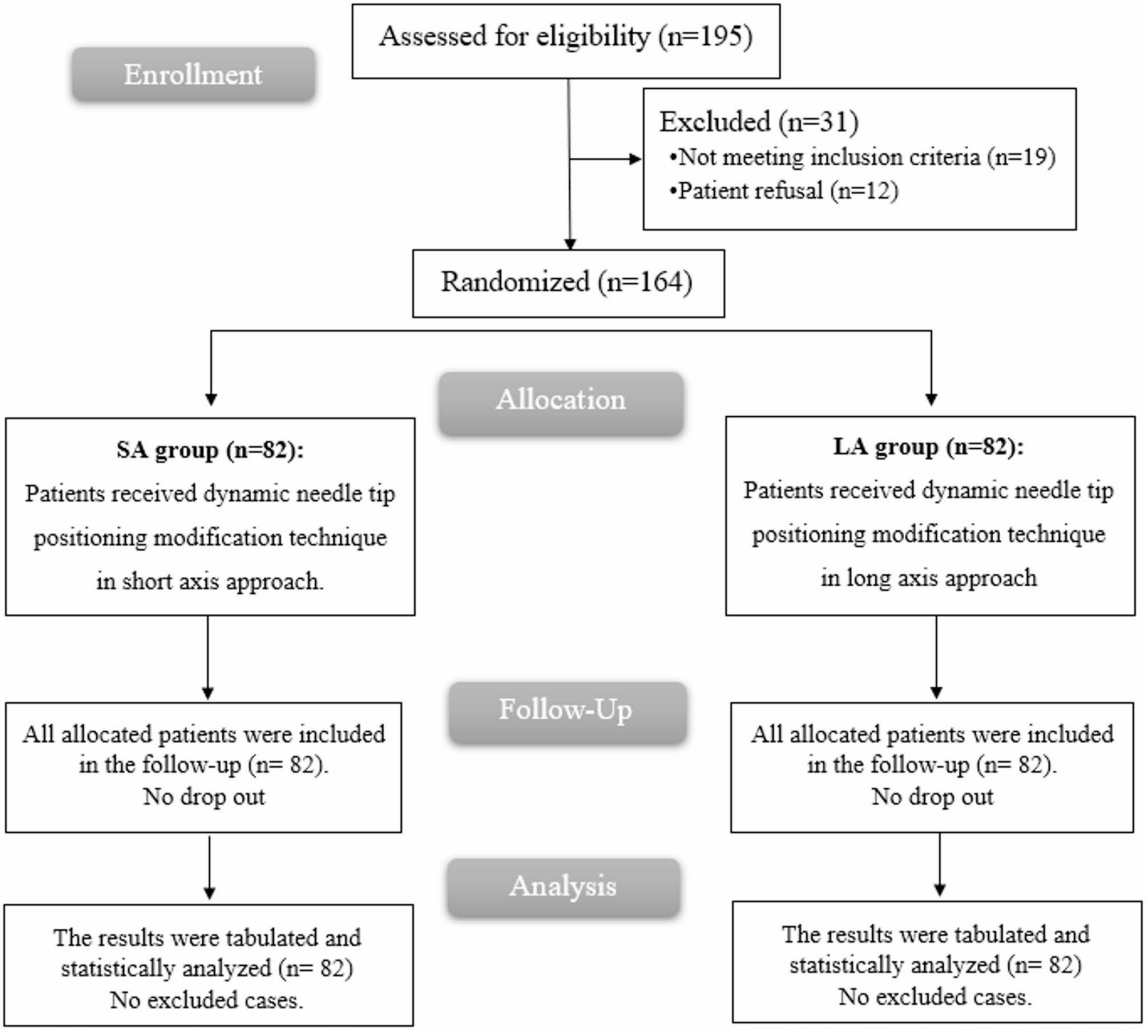
CONSORT flowchart of the enrolled patients

In this investigation, 195 patients were evaluated for eligibility; 19 individuals were deemed ineligible based on study criteria, and 12 declined study participation. Each of the two study arms received 82 randomly assigned participants, and all patients were followed and incorporated into the statistical evaluations Fig. 1.

Age, sex, weight, height, BMI, and history of peripheral vascular disease showed no significant differences between groups (Table 1).

**Table 1:** Patient characteristics of the studied groups

		SA group (*n* = 82)	LA group (*n* = 82)	*P* value	95%CI
Age (years)		46.51 ± 14.07	44.62 ± 15.27	0.411	−2.64 : 6.42
Sex	Male	59 (71.95%)	54 (65.85%)	0.399	0.68:2.58
	Female	23 (28.05%)	28 (34.15%)		
Weight (kg)		78.18 ± 13.45	76.15 ± 10.62	0.283	−1.7 : 5.77
Height (cm)		167.23 ± 7.86	168.32 ± 7.63	0.371	−3.47 : 1.3
Body mass index (kg/m^2^)		28.2 ± 5.84	27.04 ± 4.52	0.156	−0.45 : 2.77
History of peripheral vascular disease		21 (25.61%)	26 (31.71%)	0.388	0.38:1.46

Data are presented as mean ± SD or frequency (%), CI: Confidence interval

No significant disparities were observed between the groups regarding the depth of the RA’s anterior wall from the skin or the anteroposterior diameter of the RA. Nevertheless, the SA group demonstrated a notably superior first-pass success rate than the LA group (*P* = 0.022). Moreover, the SA group exhibited considerably shorter durations for both US localisation and successful cannulation than the LA group (*P* < 0.001) Table 2.

**Table 2:** Procedure data of the studied groups

		SA group (*n* = 82)	LA group (*n* = 82)	*P* value	95%CI
Depth of anterior wall of radial artery from skin (mm)		2.24 ± 0.85	2.02 ± 0.86	0.103	−0.04 : 0.48
Antero-posterior diameter of radial artery (mm)		1.49 ± 0.39	1.58 ± 0.45	0.177	−0.22 : 0.04
Ultrasonic location time (sec)		6.16 ± 1.99	13.65 ± 3.71	**< 0.001**	−8.41 : −6.57
Time to achieve successful cannulation (sec)		10.41 ± 3.25	29.09 ± 6.01	**< 0.001**	−20.16 : −17.18
First-pass success rate		76 (92.68%)	66 (80.49%)	**0.022**	1.14:8.3
Number of skin punctures	1 st attempt	77 (93.9%)	66 (80.49%)	**0.028**	0.99:1.07
	2nd attempt	5 (6.1%)	14 (17.07%)		
	3rd attempt	0 (0%)	2 (2.44%)		

Data are presented as mean ± SD or frequency (%), CI: Confidence interval

Systolic blood pressure, diastolic blood pressure, and heart rate were insignificantly different before and after RA puncture between both groups (Table 3).

**Table 3:** Vital signs before and after radial artery puncture of the studied groups

	SA group (*n* = 82)	LA group (*n* = 82)	*P* value	95%CI
Systolic blood pressure (mmHg)				
Before	122.98 ± 7.36	123.98 ± 5.7	0.332	−3.03 : 1.03
After	104.73 ± 7.97	103.34 ± 6.1	0.212	−0.8 : 3.58
Diastolic blood pressure (mmHg)				
Before	75.07 ± 6.42	73.26 ± 7.32	0.093	−0.31 : 3.94
After	68.18 ± 6.69	66.87 ± 7.78	0.247	−0.92 : 3.56
Heart rate (beats/min)				
Before	79.28 ± 8.27	77.76 ± 9.26	0.268	−1.18 : 4.23
After	76.28 ± 8.24	74.37 ± 9.3	0.165	−0.79 : 4.62

Data are presented as mean ± SD, CI: Confidence interval

The incidence rates of hematoma, vasospasm, oedema, and ischemia did not differ notably within the groups, and neither group experienced cases of thrombosis or nerve injury. However, the SA group demonstrated a notably higher level of operator satisfaction relative to the LA group (*P* = 0.007) (Table 4).

**Table 4:** Complications and operators’ satisfaction of the studied groups

		SA group (*n* = 82)	LA group (*n* = 82)	*P* value	95%CI
Complications	Hematoma	1 (1.22%)	2 (2.44%)	1	0.04:5.56
	Vasospasm	1 (1.22%)	3 (3.66%)	0.620	0.03:3.19
	Edema	0 (0%)	1 (1.22%)	1	---
	Ischemia	0 (0%)	1 (1.22%)	1	---
	Thrombosis	0 (0%)	0 (0%)	**---**	**---**
	Nerve injury	0 (0%)	0 (0%)	**---**	**---**
Operators’ satisfaction	Very satisfied	52 (63.41%)	32 (39.02%)	**0.007**	**---**
	Satisfied	23 (28.05%)	30 (36.59%)		
	Neutral	5 (6.1%)	16 (19.51%)		
	Unsatisfied	2 (2.44%)	4 (4.88%)		
	Very unsatisfied	0 (0%)	0 (0%)		

Data are presented as frequency (%), CI: Confidence interval

## Discussion

Essential for the monitoring of blood pressure and the performance of blood gas analyses in the critically ill population, AC is a pivotal procedure enhanced by USG, with techniques such as SA, LA [17], and DNTP offering distinct advantages to improve success rates and reduce complications [13].

Our study revealed significantly shorter US location and cannulation times with the SA approach (*p* < 0.001) times. These findings align with but show even more significant improvement than those reported by Mesa et al. [18], who found cannulation times of 9.29 ± 3.79 s for SA compared to 26.16 ± 20.22 s for LA. This substantial improvement suggests that our modification of the SA approach has successfully addressed some of the time-efficiency challenges.

The first-pass success rate in our SA group (92.68%) was notably higher than in the LA group (*p* = 0.022). This success rate contrasts Sethi et al. [3], who found comparable rates between SA (80%) and LA (82.6%) approaches. Our results are more closely aligned with those of Nam et al. [19], who achieved a 94% first-attempt success rate with DNTP in cardiac surgery compared to conventional LA techniques. The higher success rate in our study may be attributed to the modified technique and standardised approach.

The number of skin punctures required also demonstrated significant improvement, with 93.9% of SA cases requiring only one attempt compared to 80.49% in the LA group (*p* = 0.028). This finding is significant when considered alongside Wu et al. [20], which demonstrated that DNTP was associated with a notably higher first-attempt success rate for cannulation (pooled relative risk = 1.792, *p* < 0.001) in comparison to conventional techniques.

Our pre-and post-intervention hemodynamic parameters analysis revealed that systolic and diastolic blood pressure and heart rate measurements were statistically equivalent between groups. This adds a crucial dimension to the existing literature, as many previous studies have focused primarily on procedural success without comprehensively assessing hemodynamic impact.

The complication rates observed in our study were comparable between groups, with no significant differences in the incidence of hematoma, vasospasm, oedema, or ischemia. Notably, neither group experienced thrombosis or nerve injury. These findings contrast with the systematic review by Cao et al. [11], which found that traditional SA techniques were associated with higher rates of posterior wall puncture (RR, 3.01; 95% CI, 1.27–7.14) and hematoma (RR, 2.15; 95% CI, 1.05–4.37). Our modified SA approach appears to have successfully mitigated these previously reported risks, aligning more closely with the findings of Wu et al. [20], who demonstrated reduced complications with DNTP compared to both palpation and traditional US methods.

The superior performance of our modified SA technique can be partially explained by the findings of Sherrin et al. [21], who demonstrated a significant negative correlation between cannulation time and artery diameter (*r* = −0.602; *p* = 0.0001). The SA approach may better visualise vessel diameter and surrounding structures, facilitating more precise needle positioning. This advantage becomes particularly relevant in light of Sun et al. [22] findings regarding optimal puncture sites, which demonstrated that anatomical considerations significantly impact success rates.

While our study included a general adult population, it is worth noting the findings of Sung et al. [23] regarding elderly patients, where DNTP showed significantly higher first-attempt success rates (85% vs. 48.8%, *p* < 0.001) and shorter cannulation times (58.8 ± 22.4 vs. 89.6 ± 37.9 s, *p* < 0.001) compared to conventional LA approach. Similarly, Shim et al. [24] reported superior outcomes with DNTP in patients over 70. Our results suggest that the benefits of SA may be generalisable across age groups, though future studies specifically examining age-related variations in technique effectiveness would be valuable.

Operator satisfaction was significantly higher in the SA group (*p* = 0.007), with 63.41% reporting being “very satisfied” compared to 39.02% in the LA group. The combined “very satisfied” and “satisfied” responses (91.46% vs. 75.61%) indicate a strong preference for the SA technique. This high satisfaction rate is significant when considered alongside the findings of Oh et al. [10], who demonstrated that proficiency in DNTP techniques could be effectively developed through training. This suggests that the superior satisfaction rates observed in our study could be widely achievable with proper instruction.

The study has several limitations. It was conducted at a single centre, potentially limiting generalizability. The use of different anesthesiologists for each technique represents a significant limitation that may have contributed to the observed differences. Although patients and outcome assessors were blinded, complete operator blinding was not feasible due to the inherent differences between SA and LA approaches. The study did not assess long-term complications beyond 24 h post-procedure or evaluate the learning curve associated with each technique. External validity may be limited to elective surgical settings; emergent or hemodynamically unstable scenarios were excluded. Our findings demonstrate the efficacy of DNTP-SA in a controlled setting with experienced operators’ generalizability to novice clinicians or emergency environments.

Future studies should be multicentre, use standardised or similarly experienced operators, assess long-term outcomes, evaluate learning curves, and include emergent cases. Incorporating broader outcome measures and strategies to minimise operator bias will enhance the generalizability, validity, and clinical applicability of findings. Moreover, future studies should evaluate these techniques across varied skill levels and clinical contexts.

## Conclusions

The DNTP modification technique in the SA approach demonstrated superior outcomes compared to the LA approach for US-guided AC. SA resulted in significantly shorter US location times, faster successful cannulation, higher first-pass success rates, comparable complication rates, and higher operator satisfaction. DNTP-SA represents an efficient and safe approach for US-guided AC in clinical practice.

## Data Availability

Data is available at reasonable request from the corresponding author.

## Abbreviations

DNTP: Dynamic needle tip positioning
LA: Long-axis
RA: Radial artery
SA: Short-axis
USG-AC: Ultrasound-guided arterial cannulation
